# Early Cytokine-Induced Transient NOX2 Activity Is ER Stress-Dependent and Impacts β-Cell Function and Survival

**DOI:** 10.3390/antiox10081305

**Published:** 2021-08-18

**Authors:** Eloisa A. Vilas-Boas, Christopher Carlein, Lisa Nalbach, Davidson C. Almeida, Emmanuel Ampofo, Angelo R. Carpinelli, Leticia P. Roma, Fernanda Ortis

**Affiliations:** 1 Center for Human and Molecular Biology (ZHMB), Department of Biophysics, Saarland University, 66424 Homburg, Germany; elovilasboas@usp.br (E.A.V.-B.); christopher.carlein@uks.eu (C.C.); 2 Department of Physiology and Biophysics, Institute of Biomedical Sciences, University of São Paulo (USP), São Paulo 05508-000, SP, Brazil; angelo@icb.usp.br; 3 Institute for Clinical and Experimental Surgery, Saarland University, 66424 Homburg, Germany; lisa-nal@web.de (L.N.); emmanuel.ampofo@uks.eu (E.A.); 4 Department of Cell and Developmental Biology, Institute of Biomedical Sciences, University of São Paulo (USP), São Paulo 05508-000, SP, Brazil; davidson.almeida@usp.br

**Keywords:** β-cell, ER stress, hydrogen peroxide, insulitis, NADPH oxidase, oxidative stress, proinflammatory cytokines

## Abstract

In type 1 diabetes (T1D) development, proinflammatory cytokines (PIC) released by immune cells lead to increased reactive oxygen species (ROS) production in β-cells. Nonetheless, the temporality of the events triggered and the role of different ROS sources remain unclear. Isolated islets from C57BL/6J wild-type (WT), NOX1 KO and NOX2 KO mice were exposed to a PIC combination. We show that cytokines increase O_2_^•−^ production after 2 h in WT and NOX1 KO but not in NOX2 KO islets. Using transgenic mice constitutively expressing a genetically encoded compartment specific H_2_O_2_ sensor, we show, for the first time, a transient increase of cytosolic/nuclear H_2_O_2_ in islet cells between 4 and 5 h during cytokine exposure. The H_2_O_2_ increase coincides with the intracellular NAD(P)H decrease and is absent in NOX2 KO islets. NOX2 KO confers better glucose tolerance and protects against cytokine-induced islet secretory dysfunction and death. However, NOX2 absence does not counteract the cytokine effects in ER Ca^2+^ depletion, Store-Operated Calcium Entry (SOCE) increase and ER stress. Instead, the activation of ER stress precedes H_2_O_2_ production. As early NOX2-driven ROS production impacts β-cells’ function and survival during insulitis, NOX2 might be a potential target for designing therapies against early β-cell dysfunction in the context of T1D onset.

## 1. Introduction

Type 1 diabetes mellitus (T1D) is a multifactorial disease, in which environmental factors trigger a specific and persistent autoimmune response against pancreatic β-cells in individuals with a genetic predisposition [1]. Islets are invaded by macrophages and T lymphocytes, leading to an inflammatory response, known as insulitis, with a massive destruction of β-cells [2,3]. During insulitis, several proinflammatory cytokines, mainly interleukin (IL)-1β, tumor necrosis factor (TNF) and interferon (INF)-γ, are released by the immune cells. The main intracellular mechanisms triggered in β-cells include: (i) the activation of inducible nitric oxide synthase (iNOS) with nitric oxide (NO) production, (ii) calcium (Ca^2+^) depletion in the endoplasmic reticulum (ER) and ER stress induction, (iii) excessive generation of reactive oxygen species (ROS) and consequent oxidative stress and (iv) alteration of the mitochondrial membrane potential and activation of caspases [2,3,4]. All these mechanisms contribute to the deleterious effects on insulin biosynthesis and secretion and culminate in apoptosis.

The exposure of pancreatic islets and β-cell lines to proinflammatory cytokines, alone or in combination, leads to increased ROS production [5,6,7]. Additionally, pancreatic β-cells are believed to be highly susceptible to oxidative damage, due to a low expression of classical antioxidant enzymes [8]. However, the specific role of different sources of ROS and the time-based dynamics of ROS production in pancreatic islets during exposure to proinflammatory cytokines remain unclear. One of the main cytosolic sources of ROS is the enzyme NADPH oxidase (NOX), which is expressed in insulin-secreting cells [9,10] and whose sole function appears to be ROS production [11,12]. NOX-derived ROS play a role in the physiology of insulin secretion, but their overproduction may also be involved in β-cell dysfunction [13,14,15]. As ROS are produced and rapidly removed by intracellular antioxidant defenses, they must be particularly important in the vicinity of their production [16]. Thus, real-time assessment of the dynamic changes in ROS levels, as well as the recognition of different sources of ROS, is of great importance.

Therefore, we sought to evaluate the involvement of both mitochondrial and cytosolic sources in the dynamics of cytokine-induced ROS production and their specific involvement in the dysfunction and death of pancreatic islet cells.

The autoimmune destruction of β-cells is the hallmark of T1D and persists for years. At the time of diagnosis, around 70–80% of β-cell mass is lost; at which point, the disease is almost irreversible [2]. Understanding the early intracellular mechanisms involved in the installation of insulitis could be the key to developing specific strategies for its modulation in an attempt to preserve β-cell function and mass and prevent disease onset. Thus, we aimed to evaluate which is the main source of oxidative stress during insulitis and the temporality of the triggered events responsible for inducing β-cell dysfunction.

## 2. Materials and Methods

### 2.1. Reagents

A culture medium with or without phenol red (RPMI 1640); dihydroethidium (DHE); Fura-2 AM and proinflammatory cytokines (IL-1β, TNF and IFN-γ) from Thermo Fisher Scientific (Waltham, MA, USA); collagenase P and 4-phenylbutyric acid (4-PBA) from Sigma-Aldrich (St. Louis, MO, USA); GSK2795039 from Hycultec (Beutelsbach, Germany); a FITC Annexin V Apoptosis Detection Kit with PI from BioLegend (San Diego, CA, USA); a ViaCount kit from Merck Millipore (Burlington, MA, USA); an Insulin Ultra-Sensitive Assay kit from Cisbio (Codolet, France) and anti-p-eIF2-α (#9721S), anti-p-IRE1 and anti-α-tubulin (#3873) antibodies from Cell Signaling Technology (Danvers, MA, USA).

### 2.2. Animals

Transgenic mice expressing a genetically encoded H_2_O_2_ sensor in the cytosol/nucleus (C57BL/6J/roGFP2-Orp1) or in the mitochondrial matrix (C57BL/6N/Mt-roGFP2-Orp1) were recently generated by Tobias Dick’s group [17,18]. C57BL/6J wild-type (WT), NOX1 and NOX2-deficient (KO) mice were purchased from Jackson Laboratory (Bar Harbor, ME, EUA). Homozygous female NOX2 KO mice were crossbred with male roGFP2-Orp1 mice to obtain NOX2KO:roGFP2-Orp1. For proper heterozygous controls, C57BL/6J WT mice were crossbred with roGFP2-Orp1. All animals were kept in the animal facility of the Center for Integrative Physiology and Molecular Medicine (CIPMM) or in the animal facility of the Department of Physiology and Biophysics, Institute of Biomedical Sciences of University of Sao Paulo. Animals were maintained in collective cages (max 4/cage) at a controlled temperature (23 °C) in a 12–12-h light/dark cycle with free access to food/water. We used 10–20-week-old mice. For NOX2KO:roGFP2-Orp1 mice, only males were used. For the other experiments, both males and females were used.

### 2.3. Glucose and Insulin Tolerance Tests (GTT and ITT)

Mice were fasted for 10–12 h for GTT or 4 h for ITT. Glycemia was measured from blood collected from the tail vein at 0, 10, 20, 40, 60, 90 and 120 min after an i.p. injection of 1-g glucose/kg of body weight or 0.75-IU insulin/kg of body weight by a One Touch Ultra glucometer (Johnson & Johnson).

### 2.4. Isolation of Pancreatic Islets

The animals were randomly selected for the experiments. After anesthesia with ketamine (90 mg/kg) and xylazine (10 mg/kg), followed by decapitation, the abdomen was dissected and pancreata inflated with 4 mL of collagenase P in Krebs–Henseleit (KH) buffer (0.63 mg/mL). After full inflation, pancreata were removed and digested for 25 min at 37 °C. Pancreata were then shaken manually, and the islets were washed thrice with KH buffer (1000 rpm/5 min). Similar-sized islets were selected and randomly distributed into wells for treatments.

### 2.5. Culture of Pancreatic Islets

Isolated islets were maintained in a RPMI 1640 medium with 10% (*v*/*v*) fetal calf serum (FCS), 1% antibiotics (100-U/mL penicillin and 0.1-mg/mL streptomycin) and 10-mmol/l D-glucose, pH 7.4, 37 °C in a humidified atmosphere of 5% CO_2_ for at least 48 h before treatment. The islets were exposed to a combination of proinflammatory cytokines (10-U/mL IL-1β + 100-U/mL TNF + 14-U/mL IFN-γ) for different periods, as indicated in each figure. The concentrations of the cytokines were chosen from previous studies [5,19] and the standardization experiments from our group, using survival as the endpoint [20]. Additionally, in some plate reader experiments, the islets were exposed to 20 µM of the NOX2 inhibitor (GSK2795039) [21] or 2.5 mM of an attenuator of ER stress (4-PBA).

### 2.6. Superoxide Measurement

Batches of 30 islets from WT, NOX1 KO or NOX2 KO mice were incubated in 500 µL of KH Buffer with 50 µM of DHE dye for 20 min at room temperature. The supernatant was removed, and the islet cells were dispersed with trypsin for 2 min at 37 °C; the trypsin was then inactivated with the culture medium. The cells were pelleted (1000 rpm/1 min) and resuspended in 200 µL of culture medium. The cell suspension was transferred to a 96-well plate and fluorescence assessed by the flow cytometer Guava EasyCyte (Millipore).

### 2.7. Hydrogen Peroxide (H_2_O_2_) and NAD(P)H Real-Time Measurement

Measurements of the intracellular levels of H_2_O_2_ and NAD(P)H were performed every 10 min for 22 h, using the CLARIOstar Microplate Reader (BMG LABTECH, Ortenberg, Germany). The internal temperature was set at 37 °C and atmospheric conditions at 5% CO_2_, with open ventilation for free O_2_ diffusion in the system. Batches of 25 islets/well were transferred to U-bottom 96-well plates containing 200 μL of preheated islet medium without phenol red with or without cytokines. Cytosolic and mitochondrial roGFP2 emission in the roGFP2-Orp1 and Mt-roGFP2-Orp1 islets was detected after excitation at 405 and 488 nm and emission at 530 nm. Fluorescence from the roGFP2-Orp1 and Mt-roGFP2-Orp1 islets was normalized by subtracting the background fluorescence emitted from the WT islets (without the sensor) at the respective conditions. The results are shown as a 405/488-nm ratio. NAD(P)H autofluorescence was detected after excitation at 340 nm and emission at 450 nm, as previously described [22,23]. In addition, in some experiments, the islets were preincubated for two hours with 20-µM GSK2795039 or for five hours with 2.5-mM 4-PBA, prior to the addition of cytokines. Of note, the inhibitors were also present during the plate reader experiment.

### 2.8. Paraffin Infiltration and Sections

Islets from NOX2KO:roGFP2-Orp1 mice were cultured in the absence or presence of cytokines for 4 h and 30 min. Measurements of the intracellular H_2_O_2_ levels were made using a redox histology approach, as previously described [17,23]. Batches of 30 islets were collected and immersed in 50-mM N-ethyl-maleimide (NEM) dissolved in PBS for 20 min for sensor chemical fixation. The NEM was then removed and islets fixed in 4% paraformaldehyde for 30 min. After paraformaldehyde removal, the islets were incubated in 100-μL HepatoQuick (Roth, Karlsruhe, Germany) mixed with human citrate plasma (1:2 *v*/*v*) and 1% CaCl_2_ for 1 h at 37 °C. Clots were then incubated in 95% ethanol at 4 °C overnight and dehydrated in ethanol prior to paraffin embedding. The paraffin-embedded clots were sectioned (3-µm thick) with a semi-automated rotary microtome (Leica Biosystems, Wetzlar, Germany) and placed on silanized glass slides. Images of all the islets in each slide were obtained by the Axio Observer 7 fluorescence microscope (Zeiss, Oberkochen, Germany) with a 63x objective in both 405 nm and 488 nm. The images were analyzed with ImageJ Fiji Software, and the ratio (405/488 nm) was used to compare different groups.

### 2.9. Insulin Secretion

Batches of 10 similar-sized islets were selected and checked for the insulin secretion assay, as previously described [23,24]. Briefly, islets were incubated in KH buffer with 2.8-mM glucose at 37 °C for 30 min. The supernatant was discarded and islets were incubated at 37 °C for 60 min with a KH buffer in a low (5.6 mM) or high (16.7 mM) glucose concentration. The supernatant was collected for insulin measurements. The islets were then pooled for each condition (24 h or 48 h) and disrupted in an acid–ethanol solution (52 mL ethanol : 17 mL water : 1 mL hydrochloric acid) to obtain the intracellular insulin content. Insulin was measured by a Fluorescence Resonance Energy Transfer (FRET) using the Insulin Ultra-Sensitive Assay kit (Cisbio).

### 2.10. Ca^2+^ Measurements

After treatment, the islets from WT and NOX2 KO mice were checked for Ca^2+^ homeostasis using two protocols: (i) measurements of the total cytosolic Ca^2+^ levels and (ii) evaluation of the Store-Operated Ca^2+^ Entry (SOCE), as previously described [25]. For both protocols, the islets were incubated with 5-µM Fura-2 AM for 2 h in bicarbonate-buffered Krebs solution and placed under the fluorescence microscope Axio Observer 7 (Zeiss, Oberkochen, Germany). Measurements were made every 2 s for 20 min. For protocol (i), the islets were first incubated in a buffer without glucose, followed by the addition of high glucose (20 mM). The steepness of the curve after a glucose addition was calculated using the Gompertz equation: y = a∗exp(-exp(-k∗(x−xc))); a = amplitude; xc = center; k = steepness). For protocol (ii), the islets were first incubated in a buffer without glucose and Ca^2+^. After that, 3-μM thapsigargin was added to the empty ER Ca^2+^ stores. Finally, the islets were exposed to 3-μM thapsigargin + 2-mM Ca^2+^ to evaluate the extracellular Ca^2+^ influx by SOCE. Throughout the entire experiment, a glucose-free medium was used to avoid activation of the voltage-dependent Ca^2+^ channels. Cytosolic Ca^2+^ from individual islets was imaged using excitation at 340/380 nm and emission at 505 nm.

### 2.11. ER Stress Markers

The total protein extracts were separated by SDS-PAGE and transferred to nitrocellulose membranes. The membranes were probed with specific antibodies against p-eIF2-α and p-IRE1, using α-tubulin as the internal control, as previously described [23,26,27]. The antibodies were diluted (1:1000) in Tris-Buffered Saline with Tween buffer. One representative blot is shown.

### 2.12. Cell Death Assays

The viability was assessed by two protocols: the Guava ViaCount Reagent (Millipore), which distinguishes between viable, apoptotic and dead cells, and the FITC Annexin V Apoptosis Detection Kit with PI (BioLegend, San Diego, CA, USA). We used batches of 30 islets/condition. The protocols were performed following their respective manufacturers’ instructions, and the fluorescence was assessed by flow cytometry. For each individual experiment, the values were normalized by dividing the fluorescence of the islets exposed to cytokines by the average fluorescence of the respective untreated islets in same genotype. The values are indicated as arbitrary units (A.U.).

### 2.13. Statistical Analysis

The results are presented as the mean ± SD of at least three independent experiments, as described in the figure legends. The statistical analyses were performed in GraphPad Prism 8 software. A Student’s *t*-test was used to compare the differences between the cytokine-treated islets and respective control islets from the same genotypes. One-way ANOVA followed by Dunnett’s, Sidak’s or Tukey’s tests were used to compare multiple groups, and a two-way ANOVA + Dunnett’s test was used to compare multiple inter-related measurements between the groups, with the confidence levels set to *p* < 0.05.

## 3. Results

### 3.1. Proinflammatory Cytokines Increase the Cytosolic/Nuclear, but Not the Mitochondrial, H_2_O_2_ Levels

**Figure 1:**
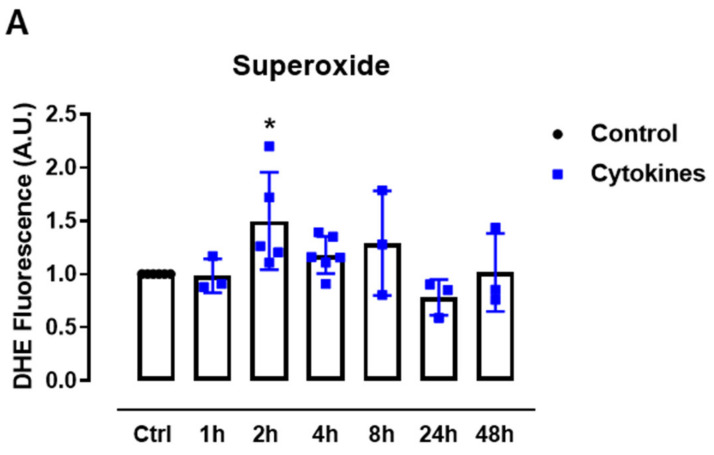

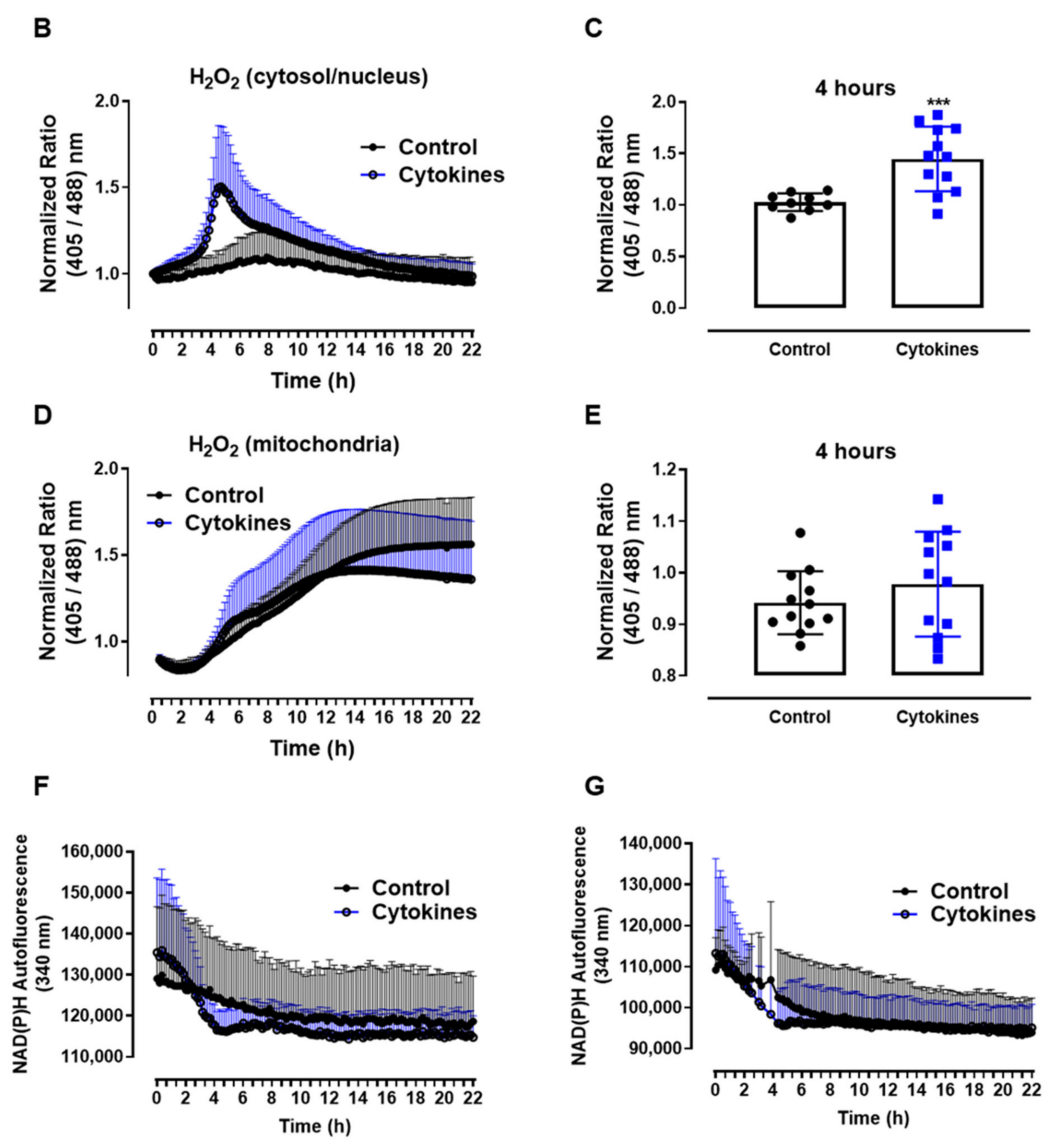
ROS production in pancreatic islets exposed to proinflammatory cytokines. (**A**) Superoxide production from wild-type islets in the absence (Ctrl) or presence of proinflammatory cytokines after different periods of exposure, as indicated. The fluorescence intensity was analyzed by flow cytometry using DHE dye. The results are expressed as the mean ± SD of 3–6 independent experiments. A.U.: arbitrary units. (**B**) Dynamic levels of the cytosolic/nuclear H_2_O_2_ of islets from roGFP2-Orp1 mice incubated with proinflammatory cytokines. (**C**) Peak of the normalized ratio of (**B**) at 4 h. (**D**) Dynamic levels of mitochondrial H_2_O_2_ of the islets from Mt-roGFP2-Orp1 mice incubated with proinflammatory cytokines. (**E**) Peak of the normalized ratio of (**D**) at 4 h. The fluorescence intensity was captured every 10 min for 22 h. The results are presented as the normalized ratio (405/488 nm) and mean ± SD of triplicates from 3 to 4 independent experiments. (**F**,**G**) Dynamic levels of NAD(P)H of the islets from roGFP2-Orp1 (**F**) and Mt-roGFP2-Orp1 (**G**) mice incubated with proinflammatory cytokines. The results are presented as the mean ± SD of triplicates from 3 to 4 independent experiments. * *p* < 0.05 and *** *p* < 0.001 when compared to the control using One-Way ANOVA + Dunnett’s (**A**) or a Student’s *t*-test (**C**). Proinflammatory cytokines mix: 10-U/mL IL-1β + 100-U/mL TNF + 14-U/mL IFN-γ.

First, we sought to systematically analyze the ROS production upon exposure to proinflammatory cytokines. Therefore, we cultured mice islets with a combination of proinflammatory cytokines based on previous studies [5,19] and monitored the static superoxide (O_2_^•−^) production at different time points by flow cytometry using the chemical probe dihydroethidium (DHE). The cytokines induced increased O_2_^•−^ production between 2 and 8 h, being statistically significant at 2 h and returning to the basal levels after 24 h of exposure (Figure 1A).

We then followed up to evaluate the H_2_O_2_ production in different cellular compartments using a state-of-art, genetically encoded redox sensor, roGFP2-Orp1, targeted at the mitochondrial matrix (Mt-roGFP2-Orp1) or the cytosol/nucleus (roGFP2-Orp1). With these sensors, we aimed to gain further insights into the source and time-based changes of ROS production. We thus isolated islets from Mt-roGFP2-Orp1 and roGFP2-Orp1 mice and exposed them to different cytokine mixes (Figure 1 and Figure S1). RoGFP2-Orp1 oxidation, reflecting changes in the H_2_O_2_ levels, was monitored in real time for 22 h under atmospheric controlled conditions using a dedicated plate reader [23,27] (see Methods). Simultaneously, the changes in intracellular NAD(P)H were also assessed by measuring its autofluorescence. After the cytokine addition, we observed increased H_2_O_2_ production in the cytosol/nucleus of islet cells between 3 and 6 h, which reached the maximum levels at 4.5 h, relative to the untreated islets (Figure 1B,C). Interestingly, the cytokines did not increase the H_2_O_2_ production in the mitochondrial matrix (Figure 1D,E) relative to the untreated islets. However, we observed that, in mitochondria, the H_2_O_2_ levels tend to increase over time, probably due to glucose usage [18,28].

Interestingly, concomitant with the peak of cytosolic H_2_O_2_ production, we observed a decrease in the NAD(P)H levels after cytokine exposure independent of the genetic background (Figure 1F,G). Other cytokine combinations (varying concentrations of IL-1β, TNF and INF-γ) also led to similar results, with small differences in the maximal effect, however (Figure S1).

### 3.2. NOX2 Pharmacological Inhibition or Genetic Deletion Abolishes Cytokine-Induced Cytosolic O_2_^•−^ and H_2_O_2_ Production

**Figure 2:**
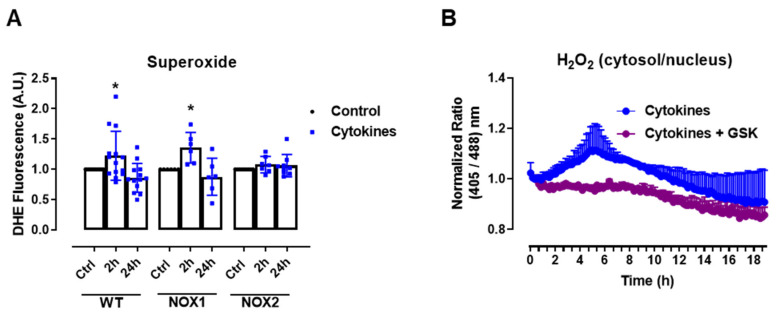

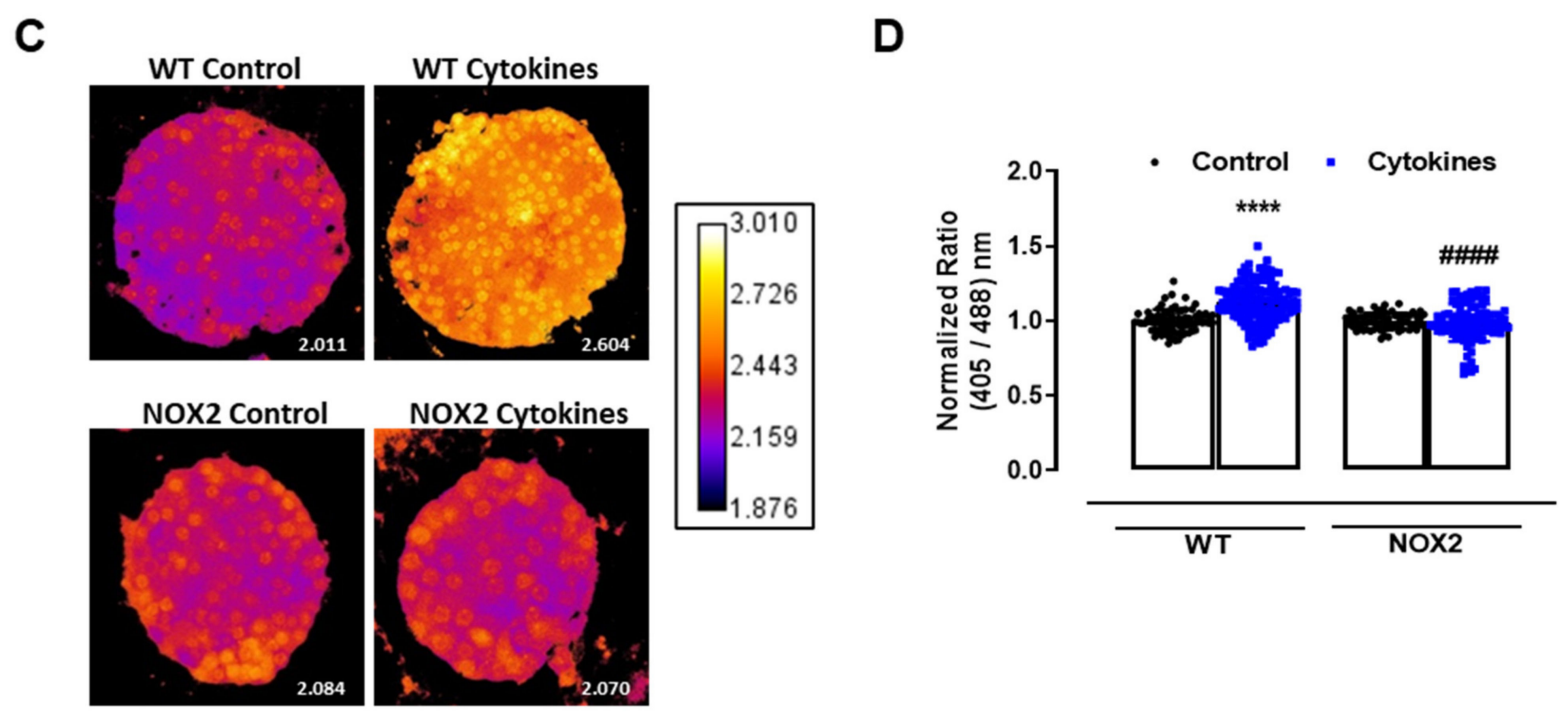
The role of NOX2 in the ROS production of pancreatic islets exposed to proinflammatory cytokines. (**A**) Superoxide production from wild-type (WT), NOX1 KO (NOX1) or NOX2 KO (NOX2) islets in the absence (Ctrl) or presence of proinflammatory cytokines after different periods of exposure, as indicated. The fluorescence intensity was analyzed by flow cytometry using DHE dye. The results are presented as the mean ± SD of 6–14 independent experiments. A.U.: arbitrary units. (**B**) Dynamic levels of cytosolic/nuclear H_2_O_2_ of the islets from roGFP2-Orp1 mice incubated with proinflammatory cytokines in the absence or presence of 20 µM of the NOX2 inhibitor GSK2795039 (GSK). The fluorescence intensity was captured every 10 min for 18 h. The results are presented as the normalized ratio (405/488 nm) and mean ± SD of 3 independent experiments. (**C**,**D**) NOX2 KO mice and roGFP2-Orp1 mice were crossbred in order to obtain NOX2KO:roGFP2-Orp1. C57BL/6J WT mice and roGFP2-Orp1 mice were crossbred in order to obtain proper heterozygous controls. The islets were incubated in the absence (Ctrl) or presence of proinflammatory cytokines for 4 h and 30 min. (**C**) Representative images of the islets from the control heterozygous (WT) and NOX2KO:roGFP2-Orp1 (NOX2) mice. The color temperatures reflect the normalized 405/488 ratio, from black/purple (non-oxidized sensor) to yellow/white (completely oxidized sensor), thus reflecting the H_2_O_2_ levels. (**D**) H_2_O_2_ levels represented as the normalized ratio (405/488). The results are expressed as the mean ± SD of individual islets from 6–9 independent experiments. (**A**) * *p* < 0.05 when compared to the Ctrl. One-Way ANOVA + Sidak. (**D**) **** *p* < 0.0001 versus the respective controls in same genotypes; #### *p* < 0.0001 versus the WT cytokines. One-Way ANOVA + Tukey’s. Proinflammatory cytokines mix: 10-U/mL IL-1β + 100-U/mL TNF + 14-U/mL IFN-γ.

The experiments above show that static O_2_^•−^ production was increased after 2–4 h of cytokine exposure in C57BL/6J wild-type (WT) islets, returning to the basal levels after 24 h (Figure 1A), which was paralleled by the H_2_O_2_ measurements with roGFP2-Orp1. As there were no significant changes in the mitochondrial H_2_O_2_ production in cytokine-treated islets, our data suggests that the cytosol is the main source of ROS in cytokine-induced oxidative stress. One of the main sources of cytosolic O_2_^•−^ and H_2_O_2_ are the NADPH-oxidases (NOX). Therefore, we aimed to investigate the contribution of two important NOXs for cytokine-induced O_2_^•−^ production, using NOX1 KO and NOX2 KO islets. Interestingly, we observed that the genetic deletion of NOX2, but not of NOX1, prevents a cytokine-induced increase in O_2_^•−^ (Figure 2A).

This suggests that NOX2 is the main NOX isoform responsible for O_2_^•−^ production upon cytokine exposure. To evaluate whether the absence of NOX2 influences the cytosolic H_2_O_2_ production, as it prevents O_2_^•−^, we cotreated the roGFP2-Orp1 islets with cytokines and a specific NOX2 inhibitor, GSK2795039 (GSK) [21]. We observed that the inhibition of NOX2 prevents a cytokine-induced cytosolic H_2_O_2_ increase (Figure 2B). To confirm this data, we crossbred NOX2 KO mice with mice expressing roGFP2-Orp1, generating the transgenic mice NOX2 KO:roGFP2-Orp1, in which NOX2 is deleted and the roGFP2-Orp1 sensor is expressed. As these mice are heterozygous for roGFP2-Orp1, the fluorescence levels are not optimal for real-time monitoring in the plate reader, which requires strong sensor expression [29]. Thus, we performed a redox histology approach, previously designed by our group [17,23] and used by others [30,31], which has been shown to reflect in vivo changes in the H_2_O_2_ levels. We exposed islets to cytokines for 4 h and 30 min, since the maximum peak of cytosolic H_2_O_2_ production was observed at this time in the real-time assessments (Figure 1B). Consistent with our real-time experiments, we observed here that cytokines increase the cytosolic/nuclear H_2_O_2_ levels in WT islets (Figure 2C,D). Consistent with the O_2_^•−^ measurements in NOX KO islets (Figure 2A) and with the H_2_O_2_ real-time measurements in GSK-treated islets (Figure 2B), the cytokines did not increase H_2_O_2_ production in the NOX2 KO:roGFP2-Orp1 islets (Figure 2C,D).

In summary, both NOX2 pharmacological inhibition and genetic ablation leads to lower cytosolic O_2_^•−^ and H_2_O_2_ production upon the cytokine treatment, implicating NOX2 in cytokine-induced oxidative stress.

### 3.3. Absence of NOX2 Is Beneficial against Cytokine-Induced β-Cell Dysfunction and Death

**Figure 3:**
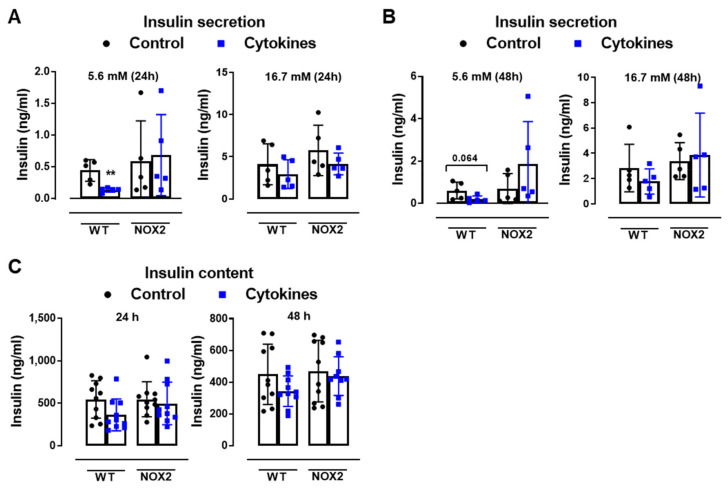

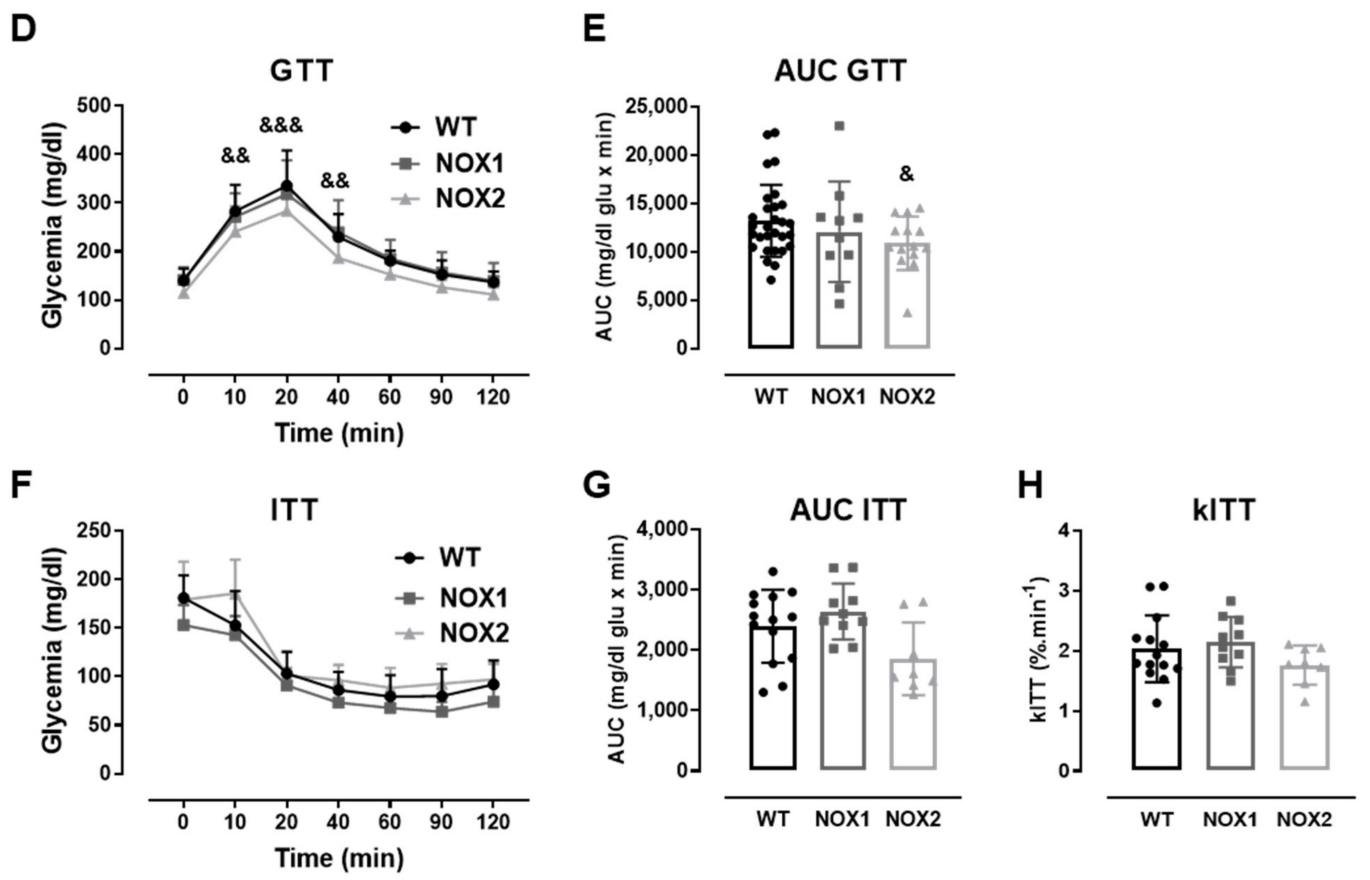
The role of NOX2 in islet function in vivo and ex vivo. (**A**,**B**) The insulin secretion of islets from WT and NOX2 KO (NOX2) islets exposed to a proinflammatory cytokine mix (10-U/mL IL-1β + 100-U/mL TNF + 14-U/mL IFN-γ). The islets were cultured in 10-mM glucose for 24 (**A**) or 48 h (**B**) and then incubated with 5.6 or 16.7 mM of glucose for 1 h. The results are expressed as the mean ± SD of 5 independent experiments. ** *p* < 0.01 versus the respective control of the same genotypes using a Student’s *t*-test. (**C**) Intracellular insulin contents of the WT and NOX2 islets in (**A**,**B**). After the insulin secretion assay, the islets were pooled for each condition (24 h or 48 h) and disrupted in a solution of acid–ethanol for an analysis of the intracellular contents of insulin. The results are expressed as the mean ± SD of 5 independent experiments. (**D**,**E**) The Glucose Tolerance Test (GTT) of WT, NOX1 KO (NOX1) and NOX2 KO (NOX2) mice. The animals were fasted for 10–12 h. The glycemia was then measured from the tail vein using a glucometer at time zero (0) and after the i.p. administration of glucose (1-g glucose/kg) at 10, 20, 40, 60, 90 and 120 min. (**D**) GTT curve (mg/dl). (**E**) Area under the curve (AUC) of (**D**). The results are expressed as the mean ± SD of 29, 10 and 14 animals for WT, NOX1 KO and NOX2 KO, respectively. (**F**–**H**) The Insulin Tolerance Test (ITT) of WT, NOX1 KO (NOX1) and NOX2 KO (NOX2) mice. The animals were fasted for 4 h. The glycemia was then measured from the tail vein at time zero (0) and after the i.p. administration of insulin (0.75-U insulin/kg) at 10, 20, 40, 60, 90 and 120 min. (**F**) ITT curve (mg/dl). (**G**) AUC of (**F**). (**H**) K_ITT_. The results are expressed as the mean ± SD of 14, 10 and 8 animals for WT, NOX1 KO and NOX2 KO, respectively. ^&^ *p* < 0.05, ^&&^ *p* < 0.01 and ^&&&^ *p* < 0.001 represent NOX2 versus WT using a two-way ANOVA + Dunnett’s (**D**) or a Student’s *t*-test (**E**).

To understand whether NOX2 deletion and decreased cytosolic ROS production protects islets from cytokine-induced dysfunction, we next analyzed the insulin secretion and insulin content from WT and NOX2 KO islets after 24 and 48 h of cytokine exposure (Figure 3A–C).

As expected, the cytokines decreased the insulin secretion and insulin content in WT islets after 24 and 48 h (Figure 3A–C). This was not observed in the NOX2 KO islets exposed to the cytokines (Figure 3A–C). Additionally, NOX2 KO mice had better glucose tolerance compared to WT mice, presenting statistically significant lower glycemia at early time points, between 10 and 40 min (Figure 3D), reflecting in a lower area under the curve (AUC) (Figure 3E). NOX1 mice, however, had the same profile as WT mice during GTT (Figure 3D). To investigate whether these animals were more sensitive to insulin, we performed an insulin tolerance test (ITT). We observed no differences between WT and NOX1 KO or WT and NOX2 KO mice in the glycemia curve after the injection of insulin (Figure 3F), reflected in similar AUC between the groups (Figure 3G). We also observed similar glucose disappearance rates (kITT) between the groups (Figure 3H). We, therefore, concluded that the NOX2 KO mice presented better glycemic levels, and this was not due to better insulin action in the peripheral tissues. Instead, it reflected a better secretory capacity in the islets, consistent with ex vivo experiments.

**Figure 4:**
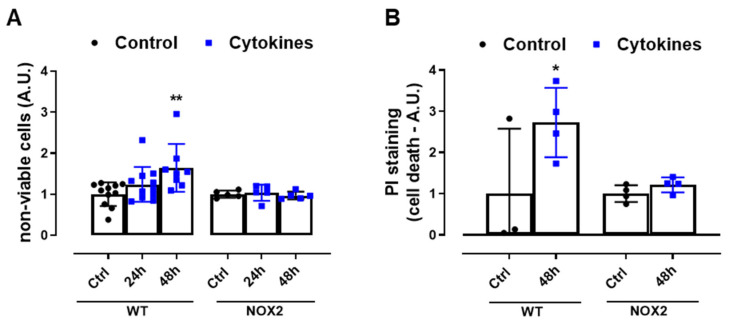
The viability of the islets from C57BL/6J (WT) and NOX2 KO (NOX2) mice exposed to proinflammatory cytokines. (**A**,**B**) The apoptosis of islets from WT and NOX2 KO mice after 24 or 48 h of incubation in 10-mM glucose in the absence (Control) or presence of a proinflammatory cytokine mix (10-U/mL IL-1β + 100-U/mL TNF + 14-U/mL IFN-γ). The apoptosis was assessed by flow cytometry using a ViaCount kit (**A**) or propidium iodide (PI) staining (**B**). For each individual experiment, the values were normalized by dividing the fluorescence of the islets exposed to cytokines by the average fluorescence of the respective untreated islets from the same genotype. The values are indicated as the fold change. The results are expressed as the mean ± SD of 5–11 (**A**) or 3 to 4 (**B**) independent experiments. * *p* < 0.05 and ** *p* < 0.01 versus the respective control of the same genotypes. One-way ANOVA + Dunnett’s.

Finally, we exposed WT and NOX2 KO islets to cytokines and monitored the cell death by flow cytometry using two different assays. The values were indicated as the fold change of the respective control (Figure 4A,B). As expected, the cytokines led to a two-fold increase in cell death in WT islets after 48 h (Figure 4A,B). In terms of percentage, this represents ~6–12% in the WT islets in the control condition and ~16–20% in the WT islets exposed to cytokines for 48 h. Although we observed that NOX2 KO islets in the control conditions had an increased basal cell death compared to WT islets (~18% in NOX2 KO islets and ~6–12% in WT islets), incubation for 48 h with the cytokines did not lead to an additional increase of cell death and, therefore, the absence of NOX2 protected from cytokine-induced cell death.

### 3.4. NOX2 Knockout Does Not Protect from the Cytokines-Induced Impairment of Cytosolic Ca^2+^ Homeostasis and Does Not Interfere with the SOCE Mechanism

**Figure 5:**
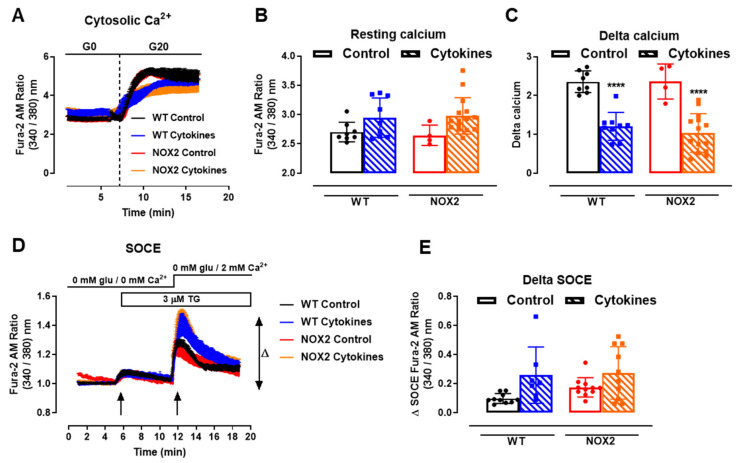
Ca^2+^ homeostasis of the islets from C57BL/6J (WT) and NOX2 KO (NOX2) mice exposed to proinflammatory cytokines. (**A**) Dynamic measurements of the total Ca^2+^ of islets from WT and NOX2 mice incubated for 24 h in 10-mM glucose in the absence (Control) or presence of a proinflammatory cytokine mix (10-U/mL IL-1β + 100-U/mL TNF + 14-U/mL IFN-γ). After incubation, the Ca^2+^ measurements were performed using Fura-2 AM dye under the microscope Axio Observer 7 for 5 min without glucose (G0) and up to 20 min with 20-mM glucose (G20). The dashed vertical line represents the addition of glucose. (**B**) Resting calcium, before the addition of glucose. (**C**) Delta, representing the response to glucose. The delta was calculated by Δ = mean_11 min_−mean_7.5 min_ in each group. The results are expressed as the mean ± SD of 4–15 individual islets (from 3 to 4 different animals per genotype). (**D**) Dynamic measurements of the Store-Operated Ca^2+^ Entry (SOCE) mechanism of islets from WT and NOX2 mice incubated for 24 h in 10-mM glucose in the absence (Control) or presence of a proinflammatory cytokine mix (10-U/mL IL-1β + 100-U/mL TNF + 14-U/mL IFN-γ). After incubation, Ca^2+^ measurements were performed using Fura-2 AM dye under the microscope Axio Observer 7 in three steps: (1) in glucose-free and calcium-free medium (0-mM glu/0-mM Ca^2+^); followed by (2) the addition of 3-μM thapsigargin (TG, first arrow), then followed by (3) the addition of 3-μM thapsigargin and 2-mM calcium-supplemented medium (0-mM glu/2-mM Ca^2+^, second arrow). (**E**) Δ SOCE as indicated in (**D**). The results are expressed as the mean ± SD of 7–14 individual islets (from 3 different animals per genotype). **** *p* < 0.0001 versus the respective control at the same genotypes. One-way ANOVA + Tukey’s.

One important step for insulin secretion is the cytosolic Ca^2+^ influx upon glucose exposure. Thus, we next checked the cytosolic Ca^2+^ dynamics in the WT and NOX2 KO islets after 24 h of cytokine exposure. Cytosolic Ca^2+^ in the WT and NOX2 KO islets in the resting state (resting calcium, Figure 5A,B) with a glucose-free buffer (G0) was similar, independent of the genotype or treatment. Subsequently, the addition of glucose at 20 mM (G20), as expected, increased the cytosolic Ca^2+^ in the WT islets, which was impaired by cytokine pre-exposure (Figure 5A,C). Surprisingly, despite NOX2 KO alleviating the cytokine-induced glucose-stimulated insulin secretion (GSIS) dysfunction (Figure 3A,B), NOX2 KO had no impact in the cytokine-induced dysfunction of the glucose-stimulated Ca^2+^ influx (Figure 5A,C). The steepness of the curve after glucose addition, calculated using the Gompertz equation, was affected by the cytokine treatment in both genotypes—1.64 and 1.57 in the untreated WT and NOX KO islets, respectively, and 0.62 and 0.79 in the cytokine-treated WT and NOX2 KO islets, respectively—thus demonstrating a slower response to glucose.

Next, we evaluated the mechanism of ER Ca^2+^ replenishment, known as store-operated calcium entry (SOCE). For that, we first applied 5-µM thapsigargin to deplete the ER Ca^2+^ stores completely in a glucose/Ca^2+^-free solution (Figure 5D, 5 min, first arrow). This was followed by the addition of extracellular 2-mM Ca^2+^ (Figure 5D, 12 min, second arrow) to provoke an extracellular Ca^2+^ influx through STIM-ORAI, as described previously [25,32]. We observed that 24 h of the cytokine treatment leads to a higher peak of Ca^2+^ in both the WT and NOX2 KO islets in a similar fashion (Figure 5D,E), indicating no participation of NOX2 in the SOCE mechanism. This also suggests that cytokines cause alterations in the ER Ca^2+^ stores, and this effect can be compensated by increasing the extracellular Ca^2+^ influx [33].

### 3.5. NOX2 Knockout Does Not Prevent Cytokine-Induced ER Stress, but ER Stress Attenuation Prevents Cytokine-Induced Cytosolic ROS

Disturbances of the ER Ca^2+^ stores are directly linked to ER stress. Indeed, several articles in the literature also show that pancreatic β-cells exposed to cytokines activate the unfolded protein response (UPR) and, depending on the exposure time and concentration of the cytokines, ultimately, ER stress. Therefore, to understand whether NOX2-dependent H_2_O_2_ production is involved in this pathway, the expression of two important UPR proteins, namely p-eIF2α and p-IRE1, was evaluated in WT and NOX2 KO islets exposed to cytokines. The islets were exposed for 4 h and 30 min, 6 h and 8 h—time points corresponding, respectively, to the maximum peak, tail end of the peak and after the peak of cytokine-induced cytosolic H_2_O_2_ production (Figure 1B).

**Figure 6:**
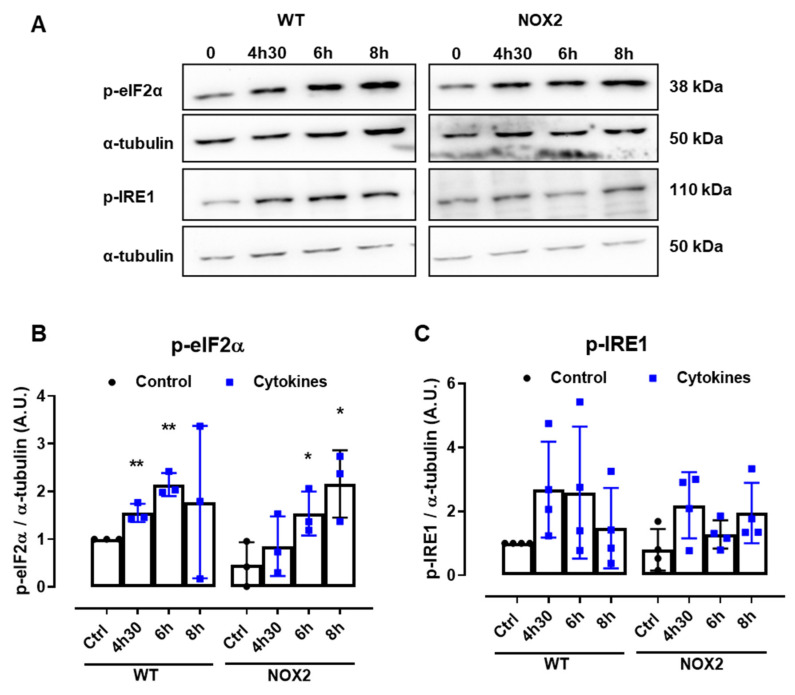

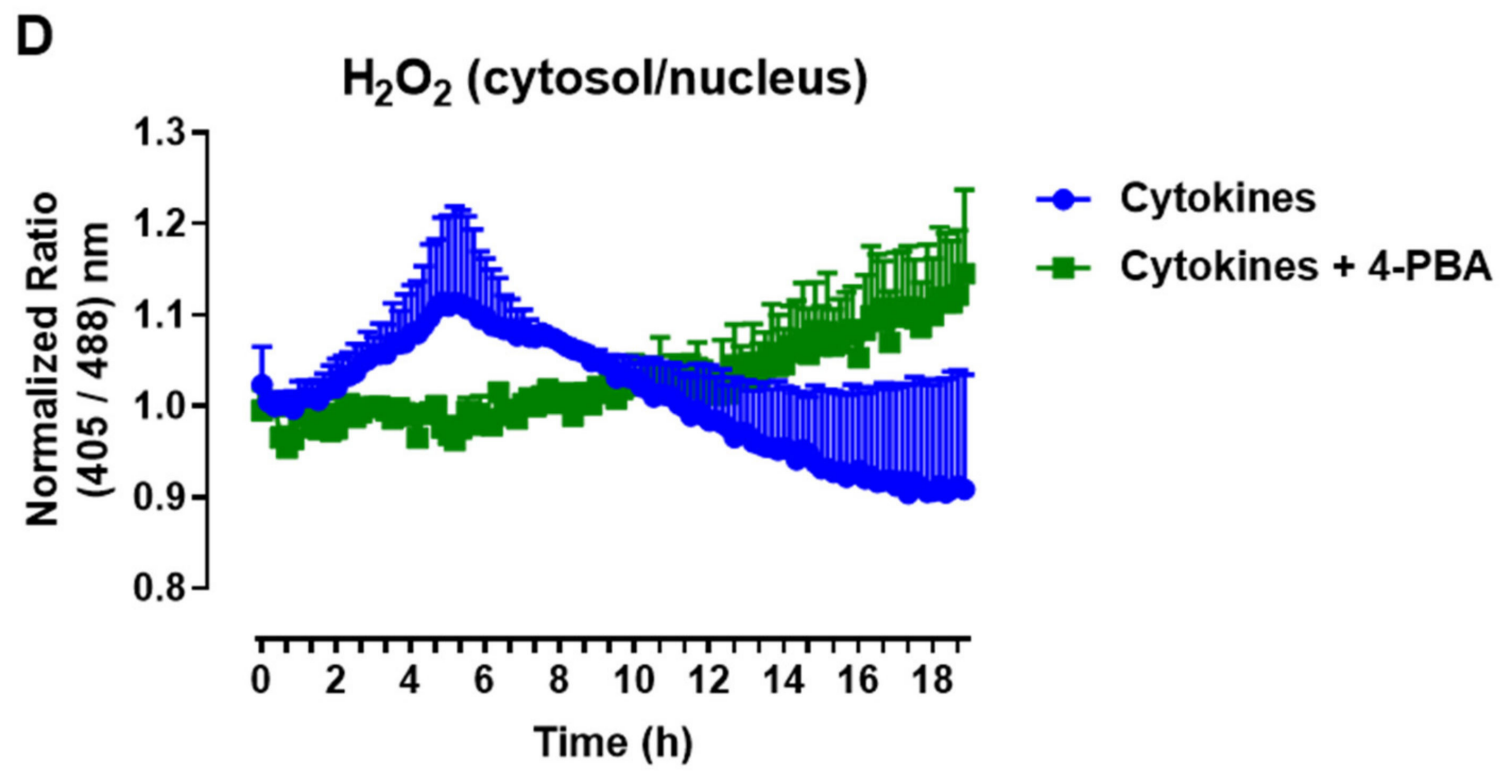
The protein expression of ER stress markers in the islets from C57BL/6J (WT) and NOX2 KO (NOX2) mice exposed to proinflammatory cytokines. The islets were incubated in the absence (Ctrl) or presence of a proinflammatory cytokine mix (10-U/mL IL-1β + 100-U/mL TNF + 14-U/mL IFN-γ) for the indicated periods. A Western blot analysis was performed for p-eIF2α and p-IRE1 using α-tubulin as the internal control. (**A**) Representative bands and the respective α-tubulin. (**B**,**C**) A densitometric analysis of the Western blot: p-eIF2α (**B**) and p-IRE1 (**C**). The results are expressed as the mean ± SD of 3 to 4 independent experiments. * *p* < 0.05 and ** *p* < 0.01 versus the respective control of the same genotype. Student’s *t*-test. (**D**) Dynamic levels of cytosolic/nuclear H_2_O_2_ in the islets from roGFP2-Orp1 mice incubated with proinflammatory cytokines in the absence or presence of the ER stress chaperone 4-PBA at 2.5 mM. The fluorescence intensity was captured every 10 min for 18 h. The results are presented as the normalized ratio (405/488 nm) and mean ± SD of 3 independent experiments.

A similar increase of both the ER stress markers was observed in WT and NOX2 KO islets exposed to cytokines (Figure 6A–C), indicating that the deletion of NOX2 has no impact on ER stress activation. Remarkably, these markers were already activated at the time point of 4 h and 30 min, suggesting that cytokine-induced ER stress precedes the cytokine-induced H_2_O_2_ peak.

To investigate this possibility, we incubated roGFP2-Orp1 islets with either cytokines alone or simultaneously with a chemical chaperone, 4-PBA, previously shown to attenuate ER stress in islets under several conditions [34,35]. The addition of 4-PBA prevented a cytokine-induced cytosolic H_2_O_2_ peak at earlier time points (Figure 6D), indicating that alleviating ER stress prevents H_2_O_2_ production and that ER stress might be an upstream event relative to NOX2-derived H_2_O_2_ upon cytokine exposure.

## 4. Discussion

ROS have been associated with proinflammatory cytokine-induced β-cell dysfunction and death in the context of T1D [36,37,38]. Here, we show that, in pancreatic islets, proinflammatory cytokines induce an increased O_2_^•−^ production and, consequently, a transient increase of the cytosolic/nuclear H_2_O_2_ levels, which are NOX2-dependent. We also show the role of NOX2 as a negative regulator of insulin secretion and viability upon cytokine exposure at later time points. Finally, we show that the inhibition of NOX2 does not affect cytokine-induced ER stress, and, conversely, cytokine-induced NOX2-derived ROS seems to be modulated by ER stress.

ROS are the key regulators of GSIS [13,14,39]. However, proinflammatory cytokines can exacerbate the ROS production in β-cells, overwhelming antioxidant defenses and leading to decreased insulin gene expression, the impairment of insulin secretion and cell death [14]. Given the importance of ROS as signaling molecules for β-cell functions and for their demise in T1D, for therapeutic purposes, the modulation of specific sources of ROS should be prioritized over attempts in ablating ROS production altogether.

To design these therapies, it is important to better understand the regulation of ROS production regarding its localization, source and timeframe. Various studies in pancreatic islets or β-cell lines have shown conflicting results regarding the proinflammatory cytokine modulation of different types of ROS production [6,7,40,41,42]. Although such differences may be influenced by the use of different models, other factors might contribute to the discrepancies among these studies, such as the employment of static methods for the evaluation of ROS based on endpoint measurements and the use of nonspecific redox-sensitive dyes, making it difficult to identify the specific sources and compartments of ROS production at a given moment.

To overcome these problems, we employed a novel strategy to assess the real-time modifications of the H_2_O_2_ levels in different compartments with a specific sensor [43]. We first evaluated the O_2_^•−^ production after exposure of the pancreatic islets to cytokines, and as O_2_^•−^ is rapidly converted into H_2_O_2_ by superoxide dismutase (SOD) [44], we also measured the H_2_O_2_ levels. The chronological patterns of O_2_^•−^ and H_2_O_2_ production were compatible, as O_2_^•−^ increased after ~2 h and H_2_O_2_ after ~4 h of cytokine exposure. Additionally, we were able to establish the pivotal role of the cytosol/nucleus, and not the mitochondrial matrix, as the main source of the transitory increase of ROS in β-cells exposed to proinflammatory cytokines. This is of great importance, since ROS are generally short-lived species [45] and, thus, must be relevant mainly in places close to their production.

Concomitant to the increased cytosolic H_2_O_2_, there was a decrease of the NAD(P)H levels. A primary mechanism by which H_2_O_2_ exerts its effects is via selective protein thiol oxidation, which is antagonized by reductive systems dependent on NAD(P)H [46]. Thus, we speculate whether the NAD(P)H decrease is related to its consumption by reductive enzymes, such as glutaredoxins, thioredoxins and peroxiredoxins, and whether it might be responsible for the subsequent decrease in H_2_O_2_. This would be interesting to be evaluated in future studies. Importantly, peroxiredoxins have been shown to participate in innumerous redox relays [47], which allow the transfer of oxidation to several target proteins and transcription factors [48,49,50]. This mechanism allows cells to specifically modulate signaling upon ROS production, even if this production is short-lived.

The observed cytokine-induced H_2_O_2_ increase mainly in the cytosol was indicative of the participation of the NOX enzyme, which is an important cytosolic source of O_2_^•−^. Consistent with this, previous studies showed that cytokine-induced increased ROS production was prevented in several β-cell models by means of different NOX inhibitors [36,37,38,42]. However, the use of inhibitors with variable specificities to several NOX isoforms casts significant doubt regarding the specific pathophysiological role of each isoform [51,52]. Here, we used NOX1 KO and NOX2 KO islets, and our results indicated the specific involvement of NOX2, since, only in NOX2 KO islets, the production of O_2_^•−^ in response to cytokines was completely abolished. In addition, we confirmed NOX2 involvement in the H_2_O_2_ real-time increase using both pharmacological inhibition and deletion by knockout. Our results indicated NOX2 as a potential target for protecting β-cells from oxidative stress during insulitis.

Previous studies have shown β-cell protection against the cytokine-induced impairment of the secretory capacity by the use of a NOX1/4 inhibitor [37] or NOX2 KO islets [38]. Moreover, the absence of NOX2 protected mice from β-cell demise and T1D development in a streptozotocin-induced T1D model [38]. Our results showed that NOX2 KO, and not NOX1 KO, mice were more glucose tolerant, and their isolated islets were partially protected against the cytokine-induced impairment of insulin secretion. Thus, the absence of NOX2 is beneficial for β-cell function both in vivo and in vitro. Particularly, our results showed that early cytokine-induced NOX2-derived ROS production impacts β-cell function.

A glucose-induced Ca^2+^ influx is the main trigger for insulin exocytosis [53,54]. Exposure to proinflammatory cytokines is known to induce perturbations in Ca^2+^ homeostasis in β-cells [33]. We showed that, although the absence of NOX2 was beneficial for the secretory function of islets exposed to cytokines, it did not impact the cytokine-induced dysfunction of the glucose-dependent Ca^2+^ response. This finding was surprising, since we observed a better secretory profile of the NOX2 KO islets. This indicates that Ca^2+^-independent mechanisms [55,56,57] may be involved in the maintenance of insulin secretion in NOX2 KO islets exposed to cytokines.

A Ca^2+^ influx is crucial for GSIS, but its greater relevance appears to occur in the first phase (triggering pathway), while the second phase (amplifying pathway)—responsible for increasing the magnitude of the secretory response—may be independent of both the K_ATP_ and Ca^2+^ influx [55]. Although the underlying mechanisms are not entirely clear, they might involve glucose metabolism and metabolic coupling factors, such as NADPH, cyclic AMP, GTP, malonyl-CoA, long-chain acyl-CoA and glutamate [58,59]. Indeed, NOX2 ablation has been previously shown to increase insulin secretion, with no major impact in the Ca^2+^ influx and metabolism [22,60], suggesting a modulatory action at distal steps on the insulin secretion process. One of the possible targets of NOX2 could be the exocytotic machinery, which has several proteins that are redox-sensitive, such as the N-ethylmaleimide-sensitive factor (NSF) [61] and SNAP25 [62]. Therefore, it is tempting to speculate that NOX2 deletion and, consequently, decreased H_2_O_2_ production may control insulin exocytosis by oxidizing thiol groups in exocytotic proteins, acting at the last step of insulin secretion. ROS have been previously suggested as stimulators of insulin secretion; however, their specific role probably depends on the stimulus, amount, source and intracellular targets [63,64].

Previous studies have focused special attention on NADPH as crucial for GSIS amplification [65,66,67]. For instance, the intracellular addition of NADPH into β-cells enhanced insulin exocytosis [65]. The precise mechanisms involved are not completely understood but might involve the NADPH-dependent activation of sentrin/SUMO-specific protease-1 (SENP1), involved in the amplification of insulin exocytosis [67,68]. Thus, in WT islets exposed to cytokines, the NOX2-derived ROS and the consequent NAD(P)H depletion observed here might play a role in the secretory impairment. It is also plausible that cytokine-treated KO islets preserve their secretory capacity due to the absence of NOX2-derived ROS and, therefore, preserve cytosolic NADPH at sufficient levels to maintain a GSIS amplification. In addition, NOX2 mediates the electron transfer to O_2_ to produce O_2_^•−^ using NADPH as the electron donor [11,12].

The main Ca^2+^ storage compartment in mammalian cells is the ER, and several studies have shown that conditions mimicking diabetes, such as β-cell exposure to palmitic acid or cytokines, lead to the depletion of ER Ca^2+^ [3,26,69,70]. The consequence of this depletion is the induction of ER stress, leading to β-cell dysfunction and death. It has been recently shown that, after ER Ca^2+^ depletion, the SOCE mechanism is activated to replenish the ER Ca^2+^ stores through the induction of extracellular Ca^2+^ entry [25,71]. Thus, we investigated whether the absence of NOX2 rendered islet cells more sensitive to cytokine-induced impairment in the SOCE mechanism and activation of ER stress. Despite, as expected, cytokines leading to alterations in the SOCE and increasing the activation of ER stress markers in WT islets, the absence of NOX2 had no significant effect on these phenomena. Further experiments are needed to completely exclude the involvement of NOX-derived ROS in cytokine-induced ER stress. However, it is known that this process is strongly related to nitric oxide (NO) production [26,72], and thus, other reactive species, such as NO, could play a major role here.

Notably, the phosphorylation of two important UPR proteins (p-eIF2-α and p-IRE1) were already increased at 4 h and 30 min, suggesting an earlier activation. This activation precedes the H_2_O_2_ production by NOX2, as seen by our plate reader measurements. A possible mechanistic explanation is that the UPR is activated prior to NOX2 or even that it is responsible for NOX2 activation, as shown in other cell types [73,74,75]. Of note, it has been previously shown that the same UPR branch is induced earlier than 4 h and 30 min by cytokines in β-cells after only 2 h of exposure and is involved in β-cell death under these conditions [76,77]. Supporting this idea, we observed here that using a chemical chaperone to attenuate ER stress delays the cytokine-induced cytosolic H_2_O_2_ peak. The precise mechanistic details and hierarchy of these pathways is not known. However, the ER stress attenuation with 4-PBA has been shown to protect β-cell functions and/or survival in several conditions, including palmitate exposure and inflammatory insult [78,79]. We suggest that this crosstalk between ER stress and, subsequently, NOX2-derived H_2_O_2_ production at a specific and early time point is closely related to the β-cell dysfunction and death in later time points. This possibility would be extremely interesting to follow up in future studies and could explain some similarities in the cytosolic ROS response between cytokine and palmitate treatments [23], as both activate UPR and ER stress, which can then converge in NOX2 activation.

## 5. Conclusions

We provide evidence that cytokine-induced NOX2-dependent transient cytosolic H_2_O_2_ production has a great impact on β-cell function and survival, without affecting the Ca^2+^ dynamics and ER stress activation, which might be upstream of the NOX2 activation. This highlights the importance of better understanding the dynamics involved in ROS production by different sources as an extremely delicate system. We believe that this is the first step towards designing more effective therapies focusing on NOX2 modulation, which could benefit β-cell preservation in the context of T1D onset.

## Data Availability

Data is contained within the article and Supplementary Material.

## Supplementary Materials

The following are available online at https://www.mdpi.com/article/10.3390/antiox10081305/s1, Figure S1: Dynamic levels of cytosolic/nuclear or mitochondrial H_2_O_2_ in islets exposed to different combinations of proinflammatory cytokines.
