# Supplementary figures and images for: Early Cytokine-Induced Transient NOX2 Activity Is ER Stress-Dependent and Impacts β-Cell Function and Survival
Source: Antioxidants (Basel). 2021 Aug 18;10(8):1305. doi: 10.3390/antiox10081305 (PMC8389306; doi:10.3390/antiox10081305)

# Suppl. Figure S1

**A**

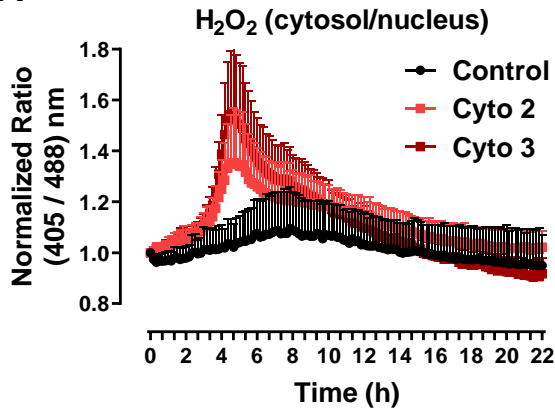

**B**

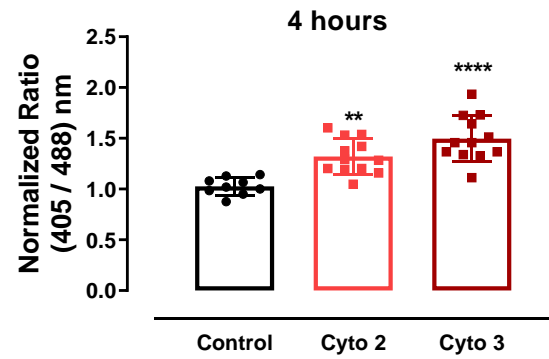

**C**

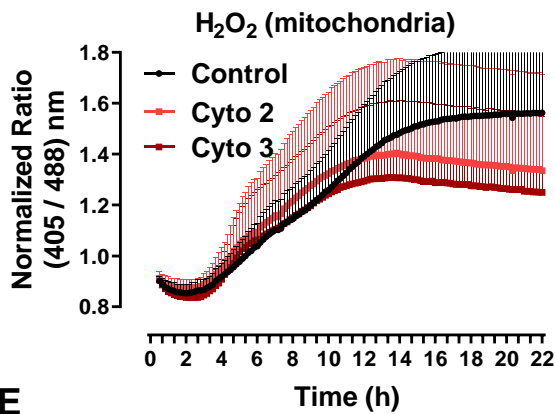

**D**

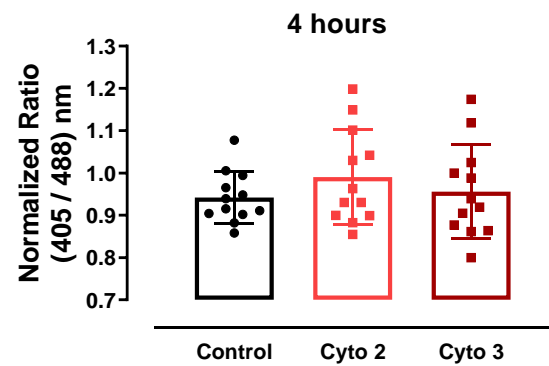

**E**

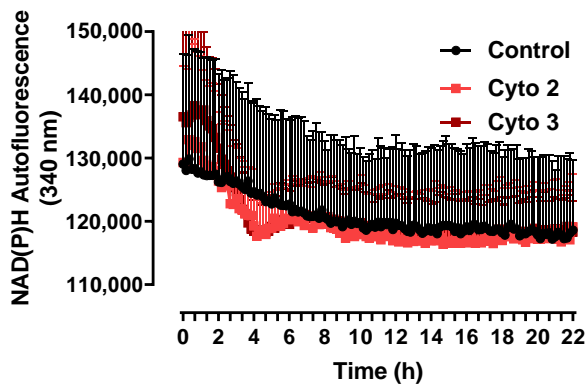

**F**

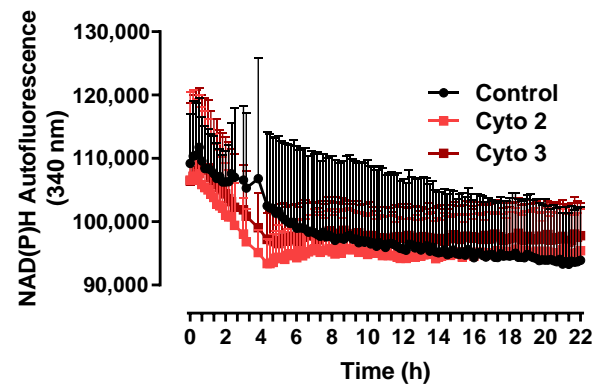

Supplement: Supplementary file 1 [file antioxidants-10-01305-s001.zip › antioxidants-1324878-supplementary.pdf]
